# An interactive dashboard for analyzing user interaction patterns in the i2b2 clinical data warehouse

**DOI:** 10.1186/s12911-024-02748-0

**Published:** 2024-11-11

**Authors:** Lena Baum, Armin Müller, Marco Johns, Hammam Abu Attieh, Mehmed Halilovic, Vladimir Milicevic, Diogo Telmo Neves, Karen Otte, Anna Pasquier, Felix Nikolaus Wirth, Patrick Segelitz, Katharina Schönrath, Joachim E. Weber, Fabian Prasser

**Affiliations:** 1 Medical Informatics Group, Center of Health Data Science, Berlin Institute of Health at Charité – Universitätsmedizin Berlin, Charitéplatz 1, 10117 Berlin, Germany; 2 Berlin Institute of Health at Charité – Universitätsmedizin Berlin, Charitéplatz 1, 10117 Berlin, Germany; 3 Charité - Universitätsmedizin Berlin, Corporate Member of Freie Universität Berlin and Humboldt- Universität zu Berlin, Center for Stroke Research Berlin, Berlin, Germany; 4 Charité - Universitätsmedizin Berlin, Corporate Member of Freie Universität Berlin and Humboldt- Universität zu Berlin, Department of Neurology, Berlin, Germany; 5 German Centre for Cardiovascular Research (DZHK), Partner Site Berlin, Berlin, Germany

**Keywords:** Health data, Clinical data warehouse, User interaction, Data visualization, Dashboard

## Abstract

**Background:**

Clinical data warehouses provide harmonized access to healthcare data for medical researchers. Informatics for Integrating Biology and the Bedside (i2b2) is a well-established open-source solution with the major benefit that data representations can be tailored to support specific use cases. These data representations can be defined and improved via an iterative approach together with domain experts and the medical researchers using the platform. To facilitate these discussions, it is important to understand how users interact with the system.

**Objective:**

The objective of this work was to develop metrics for describing user interactions with clinical data warehouses in general and i2b2 in particular. Moreover, we aimed to develop a dashboard featuring interactive visualizations that inform data engineers and data stewards about potential improvements.

**Methods:**

We first identified metrics for different data usage dimensions and extracted the relevant metadata about previous user queries from the i2b2 database schema for further analysis. We then implemented associated visualizations in Python and integrated the results into an interactive dashboard using Dash.

**Results:**

The identified categories of metrics include frequency of use, session duration, and use of functionality and features. We created a dashboard that extends our local i2b2 data warehouse platform, focusing on the latter category, further broken down into the number of queries, frequently queried concepts, and query complexity. The implementation is available as open-source software.

**Conclusion:**

A range of metrics can be derived from metadata logged in the i2b2 database schema to provide data engineers and data stewards with a comprehensive understanding of how users interact with the platform. This can help to identify the strengths and limitations of specific instances of the platform for specific use cases and aid their iterative improvement.

## Introduction

### Background

Clinical data warehouses are information systems that enable researchers to access health data for analytical purposes. They provide integrated and harmonized access to data routinely collected in healthcare settings, potentially along with research data from clinical studies, through a graphical user interface (GUI) [1]. One well-established open-source data warehouse platform is Informatics for Integrating Biology and the Bedside (i2b2), which is utilized by many healthcare institutions worldwide to facilitate the secondary use of healthcare and research data [2]. From a user perspective, the advantages of i2b2 include a GUI that is easy to use after initial training, as well as advanced mechanisms for cohort selection, including temporal queries [3]. From a data engineering and stewardship perspective, a major benefit is that i2b2 allows for flexible data representation, that can support diverse common data models [4, 5], but also customized hierarchical ontologies for specific use cases [6]. This flexible approach enables tailoring data in i2b2 to local needs and the structure and content of the local source systems [7].

In the context of clinical data warehouses, the main user group are medical researchers, who use the system for feasibility assessments, exploratory data analysis and to identify cohorts for their research projects [8]. To best support them in this process, it is the responsibility of data engineers and data stewards to define suitable data representations, ideally in an iterative approach working closely together with medical researchers [7]. By being provided with information about how users interact with the system, they can better understand how the system is being used by which user groups, which data is most relevant to the users, and how specifically the data is queried. A practical open-source solution to support this insight generation would be particularly useful as, to the best of our knowledge, there are no other existing frameworks that provide insight into user interaction patterns within i2b2 that can be deployed across different use cases and institutions. Further, this can provide a communication aid for the dialog with users to understand whether adjustments need to be made, or additional support is needed.

### Objective

The objective of the work described in this paper was to identify usage dimensions and associated metrics that are relevant for quantifying user interaction within clinical data warehouse systems in general, and for the i2b2 platform in particular. Ideally, it should be possible to derive those metrics from i2b2 metadata tables by querying the information that has been logged about user interactions. Moreover, the aim was to develop a dashboard for presenting the results using interactive visualizations. The resulting dashboard has been deployed to our local i2b2 data warehouse platform and has proven helpful in maintaining data warehouses for several user groups. The dashboard, available as open-source software, represents a practical solution for analyzing user interaction in i2b2.

## Methods

### Identification of usage dimensions and visualizations

We started by identifying data usage dimensions for software systems in general, as well as their specific equivalents for the i2b2 platform, through brainstorming and an unstructured literature review. We queried PubMed with the keywords ‘user interaction’ and ‘usage’, along with terms like ‘metric’, ‘evaluation’, or ‘quantification’, as well as ‘clinical data warehouse’ or ‘i2b2’ for use-case specific references. Our main interest was in understanding how users interact with the system. From the identified usage dimensions, we derived metrics as well as suitable visualizations. This work was performed by a team of 14 people in a two-day workshop. The workshop days were separated by a few weeks, with the second workshop building on the first. The main elements of the workshop were group discussions on relevant usage dimensions, requirements for the dashboard, and appropriate methodologies, as well as work in smaller subgroups to refine and implement prototypes of the data visualizations. Participants were medical informatics researchers with different research focuses ranging from data integration to data security and privacy, or domain experts and data stewards from one of our local i2b2 use cases. Five of the participants had previous experience with the i2b2 system through establishing and maintaining multiple data warehouse instances at a German university hospital [7, 9]. The educational backgrounds of the participants included medical informatics, computer science, engineering, and psychology.

### Metadata availability

**Fig. 1:**
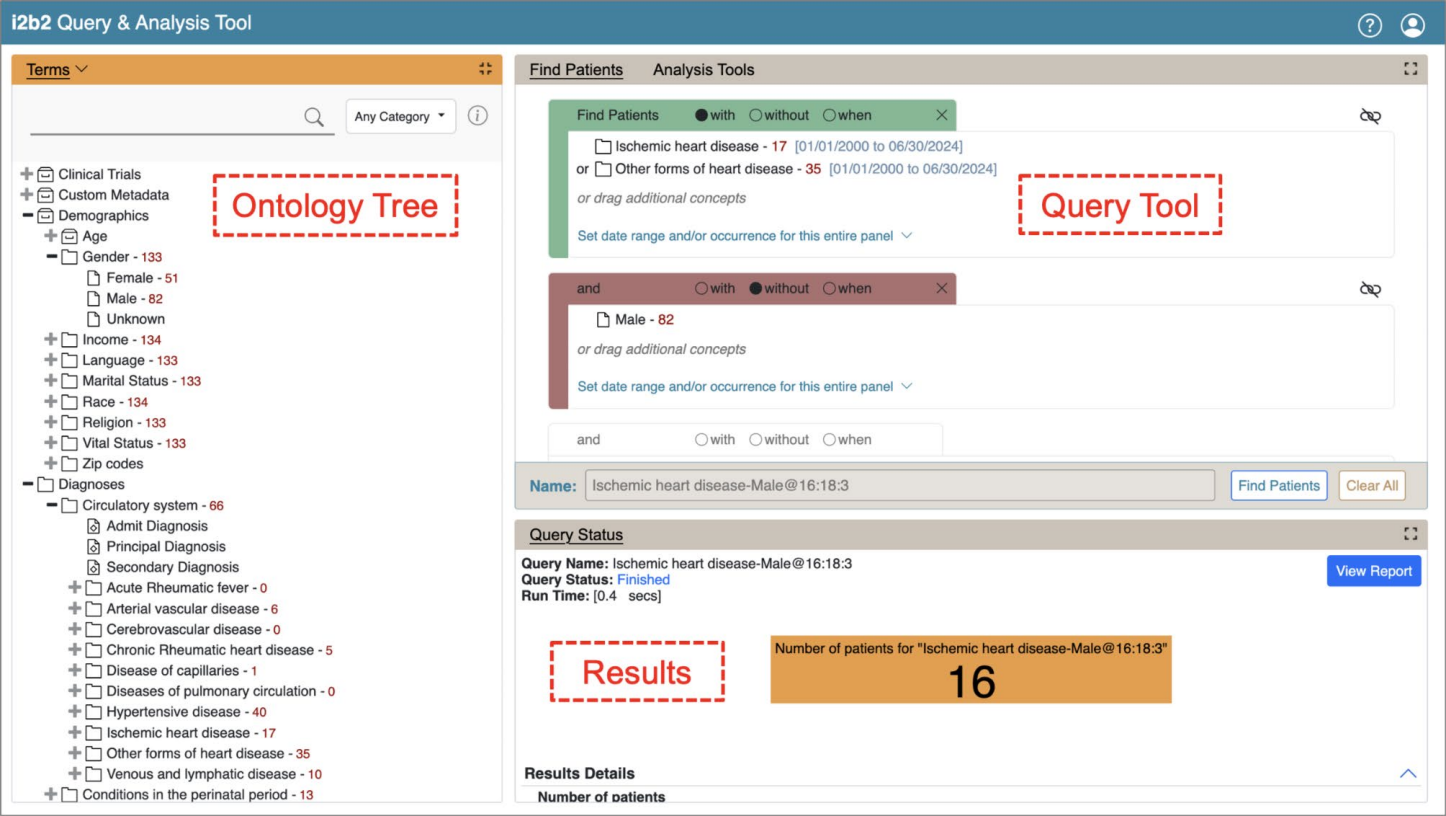
Graphical user interface (GUI) of i2b2 displaying a view of the Ontology Tree for concept selection, the Query Tool with a definition of two inclusion criteria (constrained by dates) and one exclusion criterion, and the Results section

In i2b2, the main form of user interaction with the system is by defining queries via the GUI, which is shown in Fig. 1. To define a query, users can select terms from the available ontology tree and place them in the Query Tool via a drag-and-drop mechanism. This allows users to create cohorts of patients by defining inclusion and exclusion criteria, as well as adding temporal constraints. These queries access data stored in the i2b2 star schema, which is maintained in a relational database management system, such as PostgreSQL [10], and can be accessed through the so-called Data Repository (CRC) cell [11]. This cell also stores metadata regarding previous user queries and their results. Here, important pieces of information can be derived from the Query Tool (QT) Query Master Table [12], which contains information such as the name of the query, the user ID, the creation date of the query, a request specification in Extensible Markup Language (XML) format, and the generated Structured Query Language (SQL) database query. The request XML describes the query and allows for retracing the ontology terms that users selected for their queries, with the item key listing the full hierarchical ontology path to the selected concept. The generated SQL is a transformation of the request XML, which can be executed against the i2b2 star schema.

### Implementation

**Fig. 2:**
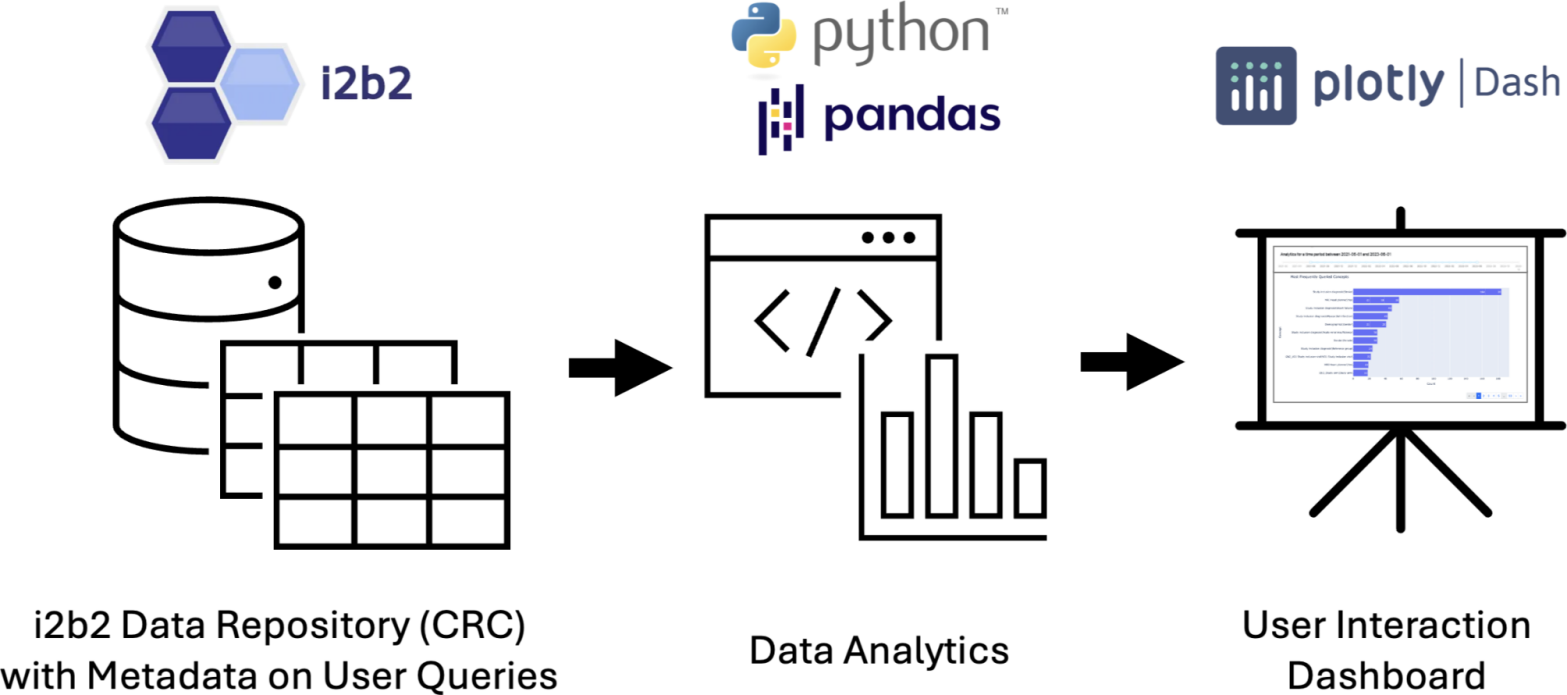
Workflow from the extraction of metadata from the i2b2 Data Repository (CRC) cell to the data analysis in Python to the final dashboard

We implemented the identified usage dimension metrics and visualizations in Python, using a variety of open-source Python libraries, such as *sqlite3* for accessing the metadata tables; *pandas*,*collections*,*itertools*,*math*, and *lxml* for data wrangling; *datetime* and *dateutil* for time-related utilities; *sqlparse* for extracting information from the generated SQL; and *plotly* for data visualizations. In a subsequent step, these visualizations were integrated into a *Dash* application. *Dash* is an open-source Python framework that builds on *plotly*,*React*, and *flask*, and supports building dashboards with data visualizations. The dashboard can thus be deployed as a standalone Python application. The process from the metadata extraction from the i2b2 Data Repository (CRC) cell [11] to the final dashboard is shown in Fig. 2. During the implementation process we used ChatGPT to get suggestions on how to use various software libraries. We reviewed and edited these suggestions as needed. To streamline the process of local deployment on our i2b2 platform and to provide the dashboard with access to the metadata tables in the i2b2 Data Repository (CRC) cell, we containerized the *Dash* application using *Docker*. In our local environment, this approach is essential, because we run multiple dockerized instances of i2b2 to support different use cases, projects and user groups [9]. This allows us to deploy an independent instance of the dashboard for each of our local i2b2 instances. To create the screenshots in the Results section, we chose one of our local i2b2 instances, featuring data from the BeLOVE (Berlin Long-term Observation of Vascular Events) study [13], a prospective cohort study focusing on cardiovascular health. In the time period analyzed we had 29 active users within this i2b2 instance. The code for the dashboard, along with information on the deployment, is openly available on GitHub [14].

## Results

### Usage dimensions

We started by identifying generic usage dimensions that are relevant to most software systems. We then applied these concepts to specific equivalents in the context of the i2b2 platform, making use of our own experience with the system as well as the i2b2 Community Wiki [15]. The results of our group discussion on usage dimensions can be found in Table 1.

**Table 1:** Generic usage dimensions and their specific equivalents within the i2b2 data warehouse system

Generic usage dimensions	Specific usage dimensions in i2b2
Number of users	User accounts created
User roles	User settings (data access user roles in i2b2) OR Types of users (e.g., clinicians, researchers, …)
Frequency of use	Number of log-ons to the system
Session duration	Time between log-on and log-off
Functionality and feature usage	Number of queries per user and/or over a period of time Frequently queried concepts Complexity of queries

An important aspect of our discussion has been the notion that usage dimensions and metrics must be interpreted in the context in which a system is being used. For example, a clinical data warehouse might be used to define data use and access requests and therefore have many users running only a limited number of queries. Other use cases might differ in usage patterns, for example, if the system is used as a data repository facilitating exploratory data analysis.

### Dashboard

Since we were primarily interested in how users interacted with the system to derive future improvements, we decided to focus on the usage-specific generic dimension of *functionality and feature usage*. By analyzing the number of queries, frequently queried concepts and query complexity, we can identify areas where users derive the most value, allowing us to prioritize improvements that enhance the user experience. This focus on queries allowed us to derive insights on i2b2-specific user interactions, as queries are the primary way of users interacting with the system. While frequency of use and session duration could offer an additional perspective, we were only able to collect login, but no logout information from the metadata, making it impossible to calculate session duration. The number of users and their specific user roles are already known to data stewards and platform administrators and are easily available through the i2b2 admin interface.

**Fig. 3:**
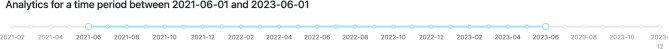
A time range slider that shows the entire period of available metadata and allows users to select the time period of interest for the analysis

For the purpose of creating a dashboard, we organized the metrics and visualizations into the following categories: (i) Queries and users, (ii) Frequently queried concepts, and (iii) Query complexity. To support studying how user interactions have developed over time a time range slider has been implemented, which is shown in Fig. 3. This makes it possible, for example, to analyze usage since the last update, e.g. when new ontologies have been introduced, to see whether and how they are being used.

### Queries and users

**Fig. 4:**
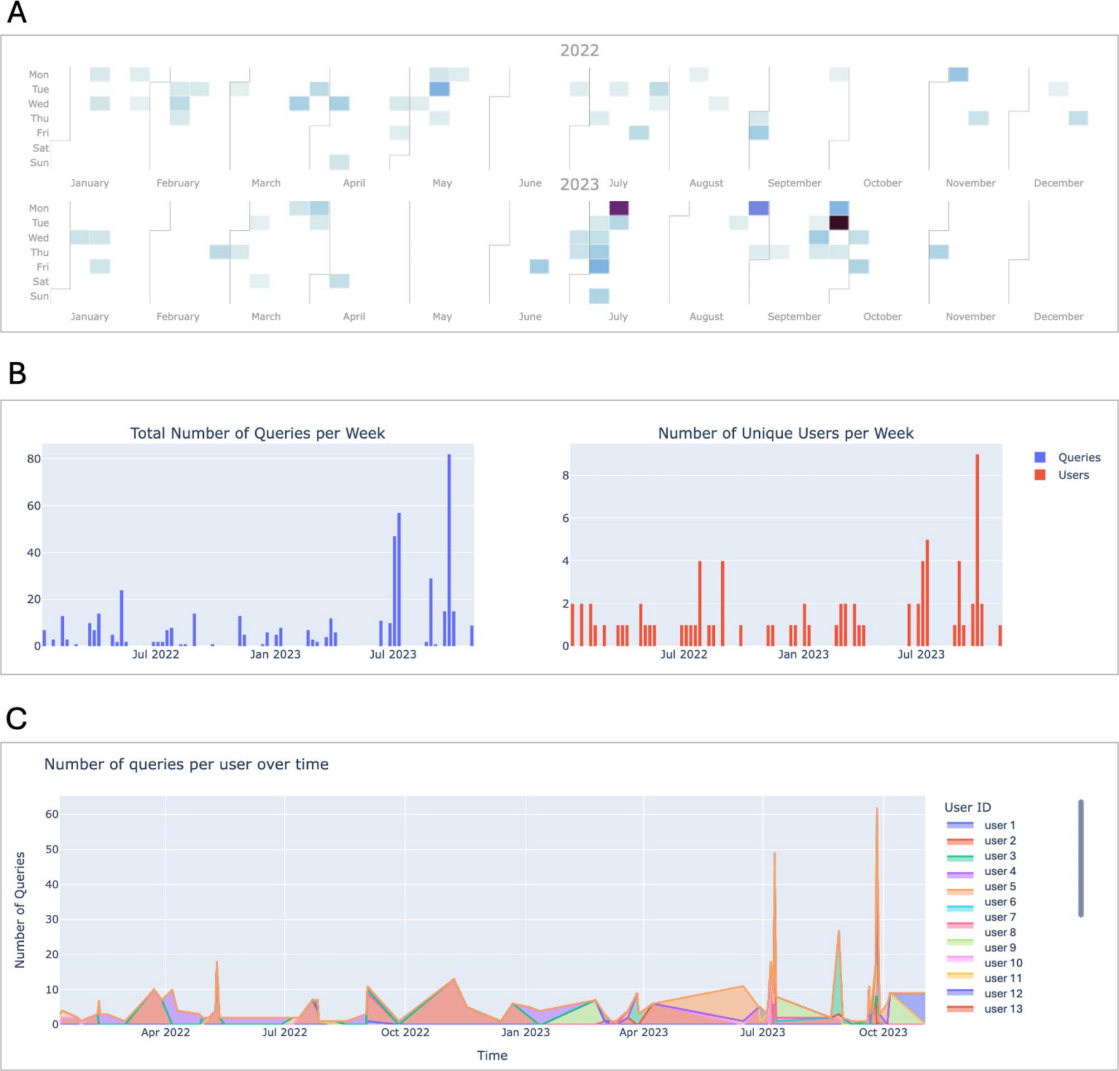
**(A)** Calendar plot showing the number of queries per day. **(B)** Bar charts of queries and distinct users per week. **(C)** Stacked area char showing the number of queries over time

To get an initial overview of the overall usage of the system, we opted for metrics and visualizations that describe the number of queries over time, along with the number of unique users that performed those queries. In addition to a tabular presentation of these numbers, we implemented a calendar plot that shows the number of queries performed per day (Fig. 4A), bar charts that show the number of queries and distinct users per week (Fig. 4B), and a stacked area chart that shows the number of queries over time (Fig. 4C).

### Frequently queried concepts

**Fig. 5:**
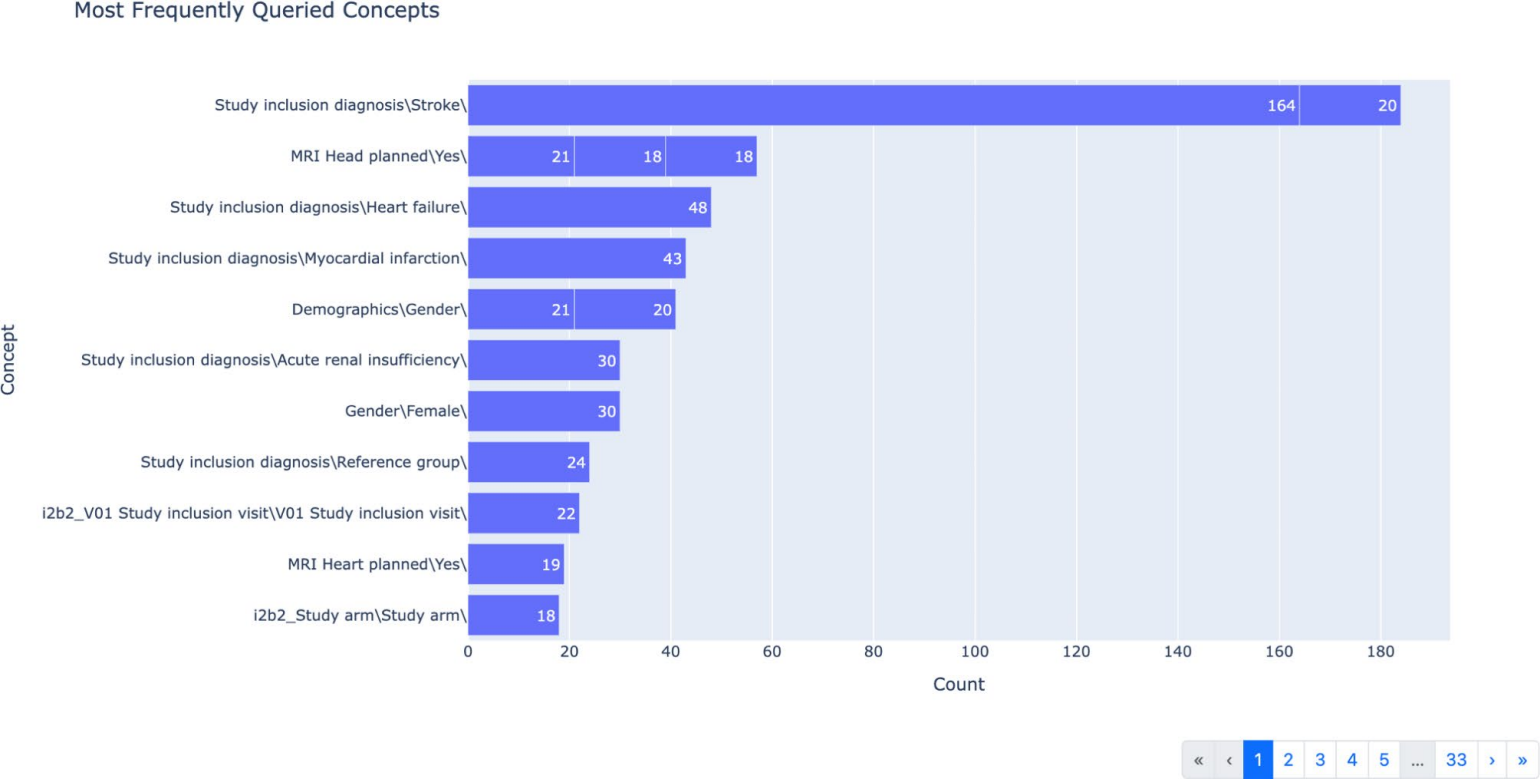
Bar chart showing the most frequently queried concepts

**Fig. 6:**
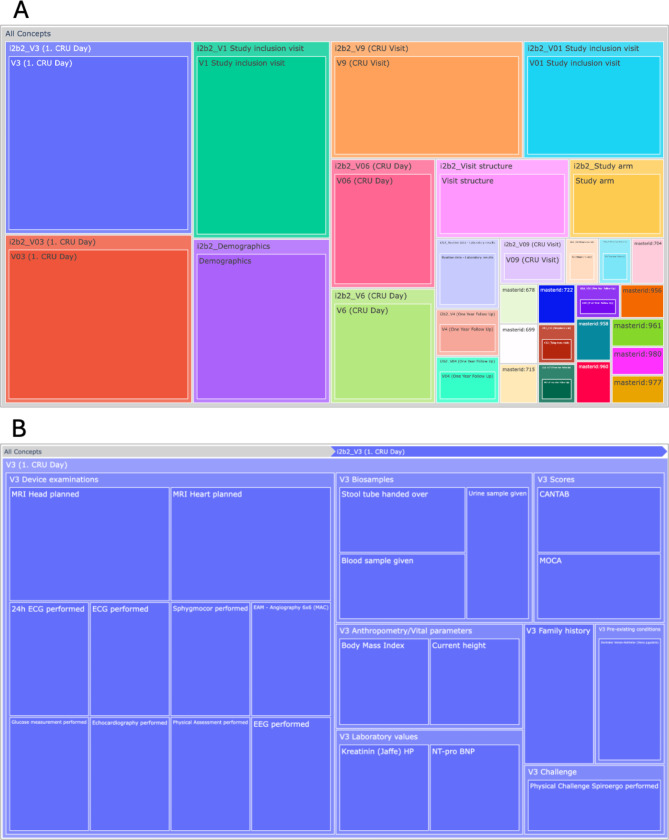
**(A)** Tree map of all queried concepts, showing up to three nested layers. **(B)** Zoomed-in view of the concepts queried of visit 3

**Fig. 7:**
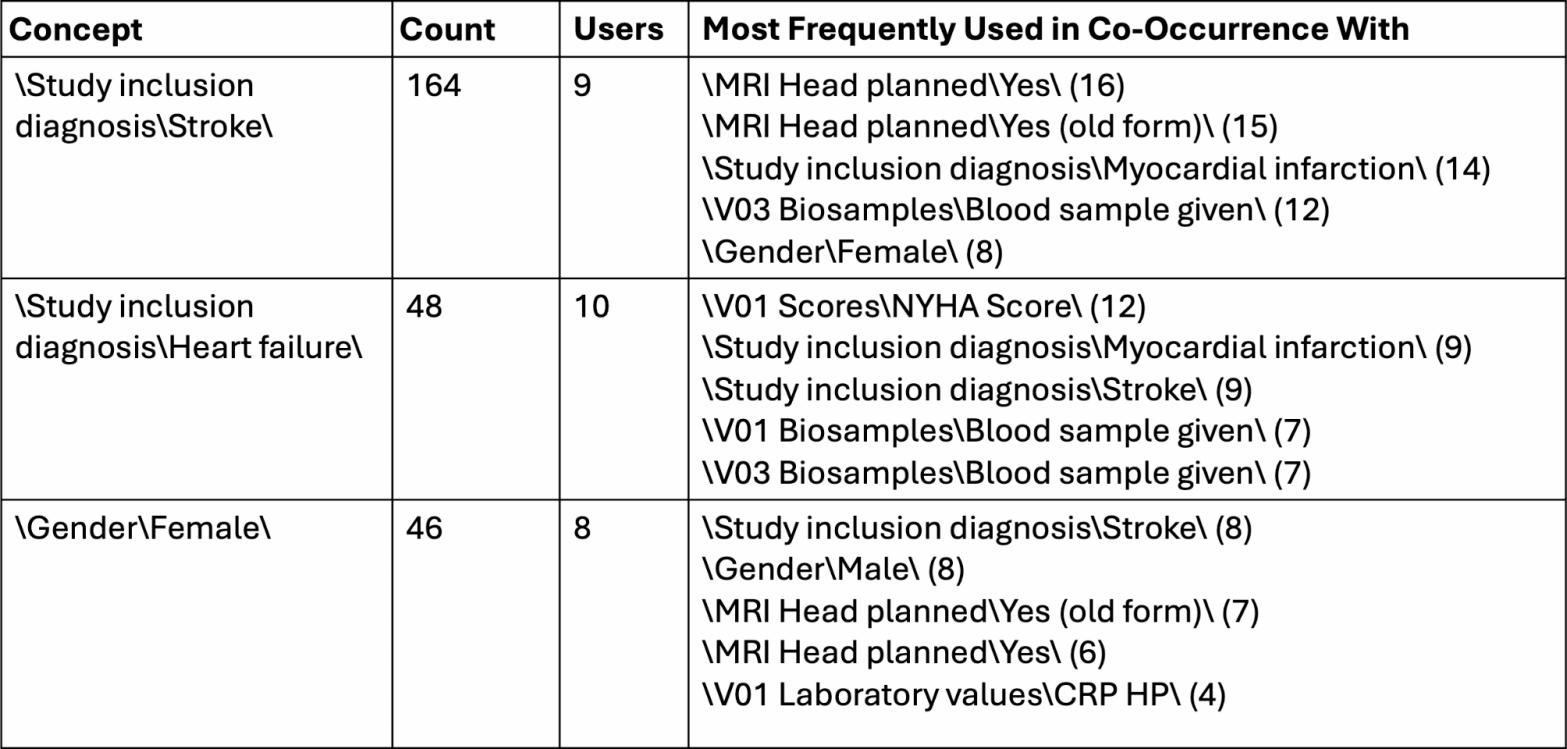
Co-occurrence table visualization, showing queried concepts, their counts, how many users they have been queried by, and the concepts they have most frequently queried in co-occurrence with, along with the respective counts

Next, we analyzed the concepts that were used in the queries to define inclusion and exclusion criteria. A bar plot visualization was chosen to show an overview of the concepts with the highest frequency (Fig. 5). For this visualization, we consider the last two elements of the full hierarchical ontology path, with the full path being displayed as a tooltip. This allows for grouping concepts that are semantically close but might exist at different locations in the ontology tree or grouping concepts for which the root of the paths might have changed over time. However, since the data is structured hierarchically, it makes sense to also include an interactive visualization that allows users to explore queried concepts within the context of the underlying hierarchy. For this purpose, a tree map visualization (Fig. 6) was implemented. Additionally, since i2b2 allows for constructing queries by combining concepts, it also useful to know which concepts are frequently queried in co-occurrence with each other. This is visualized in a table that lists concepts, their total occurrence in queries, a count of how many different users have queried the concept, and the concepts they have queried most often in co-occurrence, as well as the number of co-occurrences. An example of this co-occurrence table is shown in Fig. 7.

### Query complexity

**Fig. 8:**
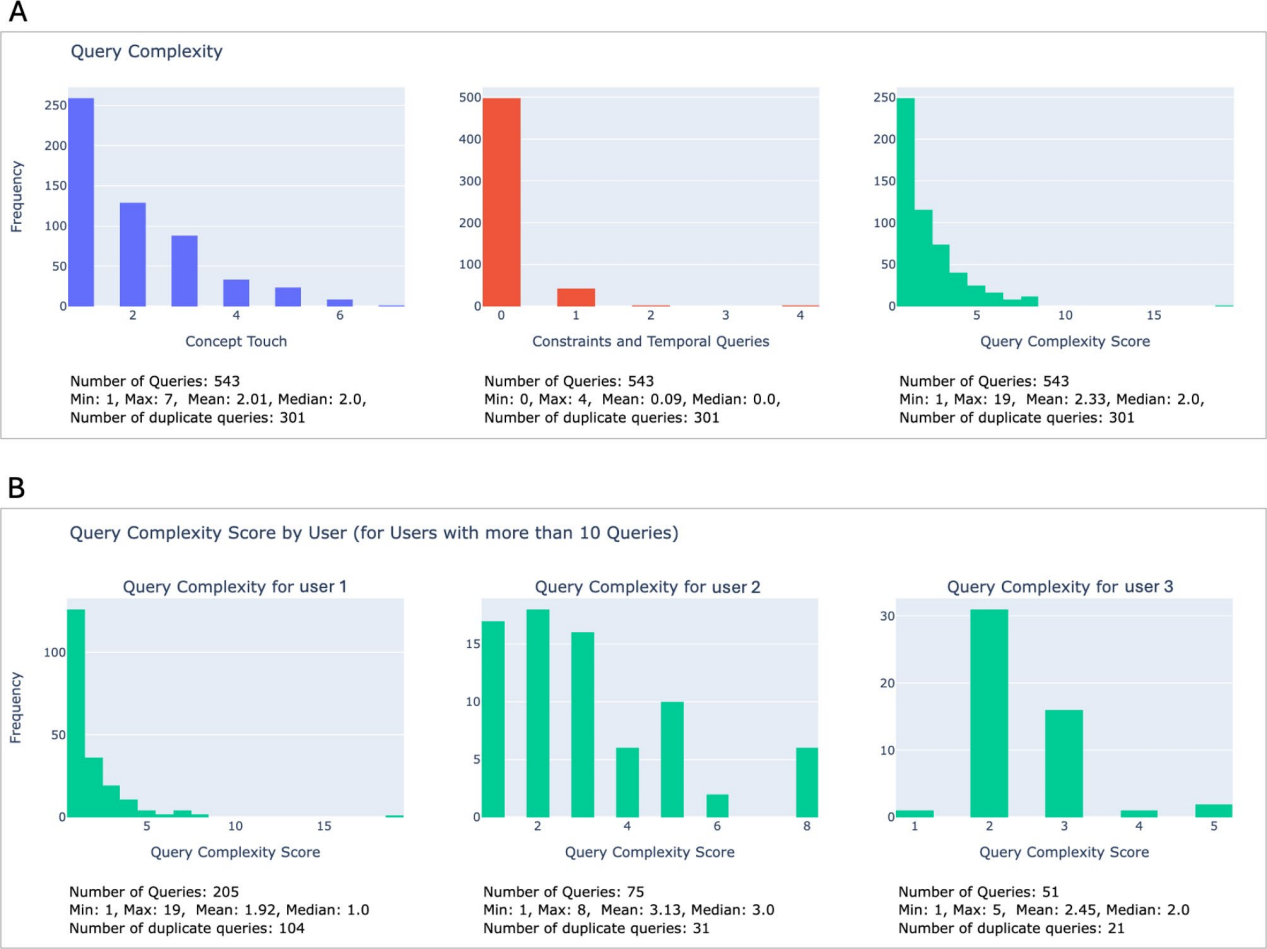
**(A)** Query complexity histograms by concept touch, constraints and temporal query counts, and combined complexity score. **(B)** Query complexity score histograms by user ID

To gain further insights into functionality and feature usage, we also analyzed the complexity of the queries. We decided to use a few easy-to-understand metrics that are closely related to the query definition mechanism in the i2b2 GUI (see Fig. 1). We opted for concepts touched (i.e., the number of ontology terms that were selected from the ontology tree and dragged-and-dropped to the Query Tool), constraints (such as value, date and total occurrence constraints, which can be selected for concepts placed within the Query Tool), and temporal query constraints (which define a temporal relationship between two events). We inferred these from the query request specifications in XML. We then combined these values into a complexity score, defined as complexity_score = (concept_touch * concept_touch_weight) + (constraints * constraints_weight) + (temporal_query_constraints * temporal_query_constaints_weight), with *concept_touch_weight = 1*,*constraints_weight = 3* and *temporal_query_constraints_weight = 5*. The weighting of the complexity score was based on the complexity of the different functionalities from a user perspective, as determined by a group discussion. However, depending on the use case, the weights might need to be adapted, which can easily be done in the code. Concept touch, constraints and temporal query constraints (as a combined count), as well as complexity scores were plotted as histograms (Fig. 8A). In addition, some basic statistical distribution measures, the overall number of queries analyzed, the minimum, maximum, mean, and median of the respective score, and the number of duplicate queries were added as annotations below. Duplicate queries can, for example, occur if the database is queried repeatedly at different periods of time as part of monitoring processes. These duplications were inferred from the generated SQL. Next, we plotted the complexity score histograms for each user who performed more than ten queries to obtain insights into different user profiles (Fig. 8B). These were annotated with the same statistical measures and duplicate query counts.

## Discussion

### User interaction insights

We deployed the dashboard to multiple of our local i2b2 instances featuring different use cases. This presented an opportunity to gain insights into actual user interaction and feature usage, other than basic information on the number of accounts created and their user roles, which are part of the i2b2 admin interface. One of the primary goals was to get a first understanding of the number of active users and the volume of queries that were initiated by those users. These numbers allowed us to quantify how much our local i2b2 instances were being actively used.

For the i2b2 instance featuring data from the BeLOVE study [13], delving deeper into frequently queried concepts, allowed us, for example, to determine that certain study visits (e.g., clinical research unit (CRU) days) were of particular interest (see Fig. 6A). Queried concepts within these visits include device examinations, biosample metadata, laboratory values, vital parameters and specific scores (see Fig. 6B). The table visualization, which provides insight into the concepts that were frequently queried together, further shows that these concepts were often paired with demographic information as well as study inclusion diagnoses (see Fig. 7). This is an expected result, as the availability of these concepts is important in the context of feasibility assessments, for which this instance is primarily being used. The analysis also revealed some initial indications of where the data representations need to be sharpened. For example, we could see that *“MRI Head planned”* and *“MRI Head planned (old form)”* were frequently queried together, which makes sense, as they semantically represent the same concept and just reflect a change in the study protocol. However, in the data representation in i2b2, there is no need for them to be distinct concepts, and they will thus be grouped together in future iterations.

Furthermore, we learned that users behave quite differently, even within the same i2b2 instance (see Fig. 8B). While some users perform queries frequently, others might have only logged in once to perform a feasibility assessment for a specific study they are planning and will then move on to a data use and access request. However, even the users who query the system frequently show different usage patterns. Some users perform many queries with low complexity (see e.g. user 1 in Fig. 8B), as they are primarily interested in getting patient counts for monitoring processes. This also results in a high number of duplicate queries. Other users tend to perform more complex queries, e.g., in a drill-down approach of defining inclusion and exclusion criteria for feasibility assessments.

One small feature that has proven particularly useful is the time range slider (see Fig. 3). This is because we update our systems iteratively, integrating new ontologies or adapting ontology representations, as well as doing frequent rounds of new user onboardings. By being able to customize the time frame for the analysis, we could get insights into whether new features are being used and how new users interact with the system.

### Related work

Despite the widespread use of i2b2 by many healthcare institutions [16], few studies have focused on how users interact with the system or collected and analyzed data usage statistics. One systematic evaluation of how users interact with the system was provided by a comparative usability study by Schüttler et al. [17]. They performed a web-based usability test that focused mainly on evaluating i2b2 as a feasibility tool, asking participants to perform predefined queries of increasing complexity. While this approach is well suited for studying usability aspects, it does not provide insight into how users actually interact with the system in a real-world setting.

Studies that evaluate the usage of i2b2 in real-word settings focus mostly on collecting basic usage statistics in the context of supporting clinical research projects within their institution. An example is provided by Lee et al. [18], who collected user characteristics and clinical data usage statistics of an i2b2-based data repository for cohort estimation and compared it to consultation by data specialists. Their usage statistics include the total number of users per month and the number of queries that look at specific clinical data categories over a period of one year. Additionally, they conducted open-ended interviews to evaluate the user experience. Another example is the study by Jannot et al. [19], who analyzed the types of research projects that were based on their clinical data warehouse over a period of five years. Moreover, Sholle et al. [20] aimed their analysis at characterizing whether medical researchers make use of complex query functionalities within i2b2. They defined complex queries as those that used more than three groups or included a temporal relationship. In addition, they analyzed which clinical domains were frequently used in the queries. Like these studies, we also analyzed users over time and assessed the concepts that were queried. However, in our approach, we have not grouped them into clinical domains. Instead, we have adopted a more generic approach that supports flexible data representations for different use cases. Additionally, none of these studies provided their metrics in an open-source framework that can be deployed across multiple institutions or a dashboard that allows data engineers and data stewards to get continuous insight into how i2b2 is being used at their institution. This practical utility of our approach is in itself a novel contribution. Other related work in the context of i2b2 are system evaluations [21–23], which include an analysis of query performance but do not include an analysis of user interactions.

To measure the query complexity, we analyzed the query request XML and opted for a similar approach to the one presented by Sholle et al. [20]. We chose a similar definition for temporal queries, which are queries that define a sequence of events. In contrast to their approach, instead of i2b2 query groups, we use concept touch, thus counting the number of concepts that were selected by users regardless of which group they define. Another difference is that we counted date, value, and total occurrence constraints placed on the selected concepts. These measures were then combined into a complexity score instead of a binary distinction between basic and complex queries.

Additionally, one could extend the scope to include the complexity of the generated SQL. While query complexity is often associated with runtime and allocated system resources, we were more interested in metrics that reflect user behavior. The idea is that combining many inclusion and exclusion criteria, as well as including temporal references, will lead to more complex SQL queries. One example of measuring SQL query complexity is provided by Moreau et al. [24], who identify metrics that determine query complexity from a user perspective. These include query length, the number of tables, attributes, subqueries, and functions, as well as the use of complex clauses. However, in i2b2, for example the number of tables and attributes touched do not provide much insight because we always have the same limited number of tables and attributes as defined by the i2b2 star schema. We thus opted for a simpler approach in which the keywords in each query were counted. We also counted temporal references since temporal constraints and temporal queries are integral aspects of i2b2. We noted, however, that the complexity scores derived from these metrics were very similar to those derived from the query XML. But since the metrics from the XML more closely mirror the actual query definitions within the i2b2 GUI, we decided to display these metrics within the dashboard.

### Limitations and future work

In our approach, we chose to focus on the evaluation of usage statistics specifically in i2b2 because it supports flexible data representations, which allows a high degree of customization but also requires additional effort to define suitable data representations tailored to the specific use case. A dashboard summarizing usage metrics can provide valuable insights that allow further refinement of these data representations. We decided to use a generic implementation that could benefit many healthcare organizations that use independent i2b2 instances for different purposes. However, due to this generic nature, the current implementation does not support, for example, characterizations of specific clinical or data domains, as these may not be applicable in each use case.

One thing we noticed when deploying our dashboard to our local i2b2 instances is that due to data representations changing over time, the same semantic concept might be represented at different hierarchical ontology paths. This can, for example, be seen in Fig. 6, where there is data available for *“V3 (1.CRU Day)”* and *“V03 (1.CRU Day)”*, referring to the same visit. This can partly be avoided if concepts are semantically grouped together by considering only the last elements of a path (as shown in Fig. 5). To address this issue systematically, we are working on incorporating provenance information that tracks changes within the data representation into the dashboard.

Our framework provides insight into the queries that users perform but not any difficulties they encounter along the way. For this purpose, additional usability evaluations are required. While traditional usability studies offer a way to test this in a controlled study setting, one way to achieve this in a real-world setting is to screen record user interactions with the system. We plan to explore this in future work, ideally in a comparative study analyzing different user groups and use cases.

Finally, we have focused only on the perspective of data engineers and data stewards that want to gain insights into user interactions, but the dashboard could be integrated into existing applications using React or extended to support further use cases. For example, the dashboard could be extended to include perspectives for system administrators, providing insight into performance, including the allocation of computing resources and memory, or helping to identify potential areas for database optimization.

## Conclusion

Different metrics covering different usage dimensions can be calculated from metadata logged by the i2b2 clinical data warehouse. Important examples include the number of queries, frequently queried concepts, and query complexity. These metrics can be visualized in an interactive dashboard to aid in the identification of strengths and potential problems of specific system instances. This information can in turn be used as a communication tool to engage in informed discussions with users and domain experts about potential improvements, for example, addressing underlying data representation issues or additional training needs.

## Data Availability

All software developed as part of this project is available as open-source at the i2b2-usage-dashboard repository on GitHub, https://github.com/BIH-MI/i2b2-usage-dashboard.

## Abbreviations

i2b2: Informatics for Integrating Biology and the Bedside
GUI: Graphical User Interface
QT: Query Tool
CRC: Clinical Research Chart
XML: Extensible Markup Language
SQL: Structured Query Language
BeLOVE: Berlin Long-term Observation of Vascular Events
CRU: Clinical Research Unit
